# Hepatitis viruses in Ethiopia: a systematic review and meta-analysis

**DOI:** 10.1186/s12879-016-2090-1

**Published:** 2016-12-19

**Authors:** Yeshambel Belyhun, Melanie Maier, Andargachew Mulu, Ermias Diro, Uwe Gerd Liebert

**Affiliations:** 1 Institute of Virology, Faculty of Medicine, Leipzig University, Leipzig, Germany; 2 School of Biomedical and Laboratory Sciences, College of Medicine and Health Sciences, University of Gondar, Gondar, Ethiopia; 3 Department of Internal Medicine, College of Medicine and Health Sciences, University of Gondar, Gondar, Ethiopia

**Keywords:** HBV, HCV, viral hepatitis, Systematic review, Meta-analysis, Ethiopia

## Abstract

**Background:**

The existing seroepidemiological data on viral hepatitis in Ethiopia showed a wide variation in prevalence pattern and the clinical and public health burden have been underestimated. The aim of this systematic review and meta-analysis was to provide a clear and comprehensive estimation of viral hepatitis epidemiology and the potential clinical burdens in Ethiopia.

**Methods:**

A comprehensive literature search was carried out from five decades (1968–2015) published studies from biomedical databases; PubMed, Google scholar, Medline and Web of Science.

**Results:**

The overall pooled prevalence of hepatitis B virus (HBV) was 7.4% (95%CI: 6.5–8.4). The pooled prevalence among subgroups showed 5.2% (95%CI: 3.7–7.4) in human immunodeficiency virus (HIV) infected individuals, 8.0% (95%CI: 5.9–10.7) in community based studies, 8.4% (95%CI: 5.4–12.7) in blood donors, 11.0% (95%CI: 7.5–15.9) in immigrants and 6.9% (95%CI: 5.6–8.5) in other groups. Among study parameters considered during meta-regression analysis, only study years were associated with a decreasing HBV prevalence rate over time. The overall pooled prevalence of anti-hepatitis C virus antibody (anti-HCV) was 3.1% (95%CI: 2.2–4.4). Unlike HBV, the anti-HCV prevalence in HIV infected individuals was higher (5.5%, 95%CI: 3.8–7.8%, *p* = 0.01) than the prevalence observed in the other subgroup of study population. Although relatively few data were available, hepatitis virus A (HAV), D (HDV) and E (HEV) were also circulated in Ethiopia.

**Conclusions:**

This review indicates that all types of viral hepatitis origins are endemic in Ethiopia. Adapting a recommended diagnostic and treatment algorithm of viral hepatitis in the routine healthcare systems and implementing prevention and control policies in the general population needs an urgent attention.

**Electronic supplementary material:**

The online version of this article (doi:10.1186/s12879-016-2090-1) contains supplementary material, which is available to authorized users.

## Background

Hepatitis viruses, such as HAV, HBV, HCV, HDV and HEV cause potentially life-threatening inflammation of the liver, which is characterized by acute and chronic forms of liver disease. According to 2013 World Health Organization (WHO) global health impact report of viral hepatitis [1], more than 240 and 150 million populations were affected by chronic liver disease due to HBV and HCV, respectively. Africa has the second largest number of chronic HBV carriers after Asia and is considered as a region of high endemicity [2]. Despite its high prevalence and highly infectious nature, HCV remains under-diagnosed and under-reported in most African countries [1]. Regarding HAV, Africa is known by high prevalence rate and nearly all older children and adults in the region acquired anti-HAV antibody immunity [3]. HEV is also one of the leading causes of major outbreaks of acute viral hepatitis worldwide, especially in developing countries [4]. Similarly, HDV is prevalent worldwide and associated with the most severe form of viral hepatitis [5].

In Ethiopia, an old clinical study showed that liver disease accounted for 12% hospital admissions and 31% hospital mortality [6]. Moreover, in Ethiopia and neighbouring Kenya more than 60% of chronic liver disease and up to 80% of hepatocellular carcinoma (HCC) are due to chronic HBV and HCV infections [6, 7]. Unlike HAV, HDV and HEV, which are not extensively studied, several HBV and HCV seroepidemiological studies were available in the country. However, the majority of the reports showed epidemiological variations of 2.1 to 25.0% over time and across geographical areas as well as the same localities [8–17]. More importantly, because of HIV pandemic and possible epidemiological overlap as the result of shared transmission ways and risk factors, viral hepatitis-HIV co-infection and subsequent severe forms of clinical complications could be potentially high in the country. Nevertheless, the clinical and public health burdens due to viral hepatitis in general are still given no emphasis in the country’s health system. For instance, a recent report showed the presence of very limited knowledge, minimal awareness and underestimation of the viral hepatitis prevalence and disease burden in the country, which have resulted insufficient budgetary and organizational focus [7]. Moreover, according to the WHO report, Ethiopia is regarded as a country with no national strategy for surveillance, prevention and control of viral hepatitis, but the country is classified under the geographical regions with intermediate to hyperendemic viral hepatitis infections [1]. Thus, the lack of a consolidated epidemiological data on the burden of viral hepatitis in Ethiopia might be responsible for the absence of practical action at the policy level. Therefore, this systematic review and meta-analysis was made using data published in the last five decades (1968–2015) to provide a quantified estimate of the problem as a step toward for a better understanding of the viral hepatitis epidemiology, clinical burden and the situation of human immunodeficiency virus (HIV) co-infection in Ethiopia.

## Methods

### Search strategy

A comprehensive literature search was carried out from biomedical databases; PubMed, Google scholar, Medline and Web of Science according to Operations Manual of the Global Burden of Diseases for systematic epidemiological reviews of targeted diseases or condition frequency [18] and Preferred Reporting Items for Systematic Reviews and Meta-analysis (PRISMA) [19]. The search focused on all published studies with epidemiological and/or clinical data on the seroprevalence of hepatitis viruses (HAV, HBV, HCV, HDV, and HEV) in Ethiopia from the first scientific description (1968) to 2015. During the search, the following keywords (terms) were used; [“hepatitis A” AND (seroprevalence OR prevalence) AND “Ethiopia”], [“hepatitis B” AND (seroprevalence OR prevalence) AND “Ethiopia”], [“hepatitis C” AND (seroprevalence OR prevalence) AND “Ethiopia”], [“hepatitis D” AND (seroprevalence OR prevalence) AND “Ethiopia”], [“hepatitis E” AND (seroprevalence OR prevalence) AND “Ethiopia”]. The search was repeated by replacing the full written phrases using the abbreviated form of each respective hepatitis virus. Similarly, key terms like “Epidemiology”, “Viral liver disease”, “Viral hepatitis and HIV co-infections” were used in place of the terms “Seroprevalence” and “Prevalence”.

### Inclusion and exclusion criteria

**Fig. 1:**
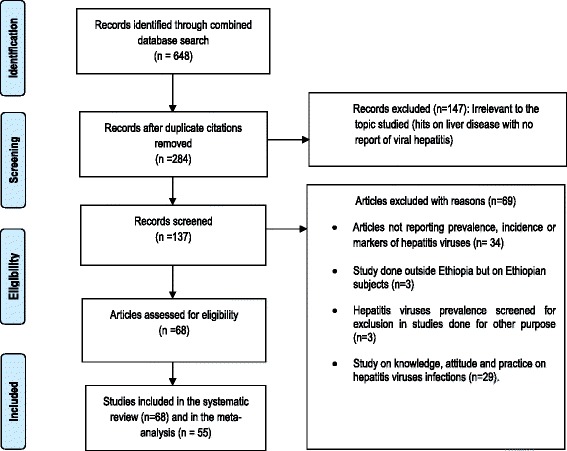
Flow diagram of studies reviewed, screened and included

Abstracts were screened to determine the relevance of each study. All studies that reported “seroprevalence” and/or “clinical isolation of viral hepatitis” were selected for inclusion according to PRISMA Flow Diagram [19] (Fig. 1). The inclusion was restricted to original research articles published in English language. The studies also included Ethiopian Jews who immigrated to Israel and screened for hepatitis viruses at the time of their arrival.

### Data source

The screened articles were organized and subgrouped into five major study population types: community based, blood donors, symptomatic liver disease patients, HIV co-infected individuals and various other groups (healthcare workers and medical waste handlers, out and inpatient department patients, street dwellers, pregnant women, diabetic patients, HIV voluntary counselling and testing (VCT) centres clients and commercial sex workers).

### Data extraction and quality assessment

In each abstract and/or full text of the article which was considered to be eligible, information about the study area, year of sample collection and publication, study population, sample size, type of screening kits used and other useful variables were recorded (Additional files 1 and 2). The studies were assessed to identify ‘seroprevalence’ as an outcome of serological evidence for each of the hepatitis viruses. The quality and eligibility of the screened articles were assessed using a 12 point scoring system based on modified Downs and Black [20] check lists. Special emphases were given for clarity of objective, method, study population characteristics and finding presentations. Articles with low quality score were excluded during the meta-analysis (Additional files 1 and 2).

### Data analysis

The extracted data were systematically restructured among viral origin, study area, study groups and study year during the systematic review. The subgrouped data were analysed using simple descriptive statistics during the systematic review. Accordingly, a median and inter quartile range (IQR) values across each subgroup (community based, blood donors, symptomatic liver disease patients, HIV infected individuals, and various other groups of the society were calculated because of expected heterogeneity in terms of the study area, time, sample size and the study design.

During the meta-analysis, all selected studies were combined and a random effects model by DerSimonian and Laird [21] were used to estimate overall pooled prevalence. Since the risks of viral hepatitis exposure are potentially high in symptomatic liver disease patients, the meta-analysis was done independently in order to avoid the potential bias on the outcome of pooled estimation. Cohran’s Q (low *P* value indicates presence of heterogeneity) and and I squared (I^2^) (with I^2^ > 50% denoting substantial heterogeneity statistical tests) were conducted to test heterogeneity [22]. The subgroup analysis was performed among the community based studies, blood donors, HIV infected individuals and other groups. The subgroup analysis was not considered for the screening kits used because of the observed homogeneity in the types of the kits (which include an immunoassay based screening kits such as enzyme linked immunoassay, radioimmunoassay and chromatographic immunoassay) (Additional files 1 and 2). Meta-regression analysis was used to determine potential confounders such as mean age groups, study population types, year of study and geographical zones. The mean age group meta-regression was considered for those studies which only reported the mean age (Additional files 1 and 2). The overall fixed and random effects model with 95% confidence intervals (95% CIs) were calculated and illustrated using a forest plot graph presentation. The meta-analysis was not considered for data extracted for HAV, HDV and HEV since the number of available studies was very small. Compressive meta-analysis software version 3.3., 2014 (www.meta-analysis.com) was used during the meta-analysis.

## Results

### Demographic and study population characteristics

The population of Ethiopia was 22 million at the time (1960s) when the first HBV seroprevalence data were reported. After 30 years, when many of the seroepidemiological and few clinical reports of hepatitis viruses were available, the population steadily increased to 48 million [23]. Currently, with a population of 94 million, the country is the second most populous in Africa. The total study population size screened for hepatitis viruses and involved in this systematic review and meta-analysis were 79,931. Among these, 62,955 were screened for hepatitis viruses from the general population. About 5,229 were from symptomatic patients with acute (867) and chronic (1020) liver diseases, and outpatient department attendants (3,342). The rest 11,747 were from HIV infected individuals. Geographically, the majority of population screened for hepatitis viruses were from central Ethiopia (mainly in Addis Ababa) (45,037), northern Ethiopia (16,071), southern Ethiopia (17, 207) and from Ethiopian immigrants to Israel (1616). Overall, 68 studies were eligible for the review and among these, 26 studies reported both HBV and anti-HCV seroprevalence and the rest 6 and 36 studies reported only HCV and HBV, respectively. The mean age group of the study population screened for HBV and HCV was 28.9 ± 6.1 (range 14–48) and 28.2 ± 7.3 (range 16–55) years old, respectively. The peak prevalence was reported between the group of 24–39 and 20–37 years old, respectively to HBV and HCV (Additional files 1 and 2). All of the selected studies were used immunoassay based kits of various types as their primary screening test. In addition, only four (6.1%) studies (two each for HBV and HCV) used PCR for measuring viraemia level (Additional files 1 and 2).

### Epidemiology of HBV

**Fig. 2:**
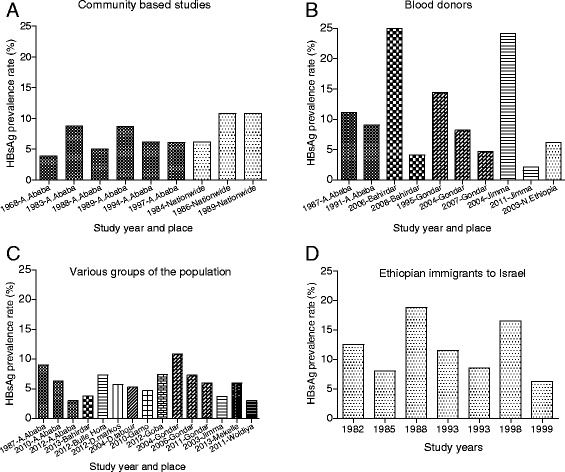
HBsAg prevalence among the community based studies (**a**) [24–26, 52, 59, 76, 85, 87, 88], blood donors (**b**) [8, 9, 13–17, 57, 89, 90], various groups of the society (healthcare workers, outpatient department attendants, medical waste handlers, pregnant women, HIV voluntary counseling and testing (VCT) clients, commercial sex workers) (**c**) [10, 29–41] and Ethiopian Jews immigrants to Israel (**d**) [54, 91–96]

The first documented HBsAg prevalence rate was 3.9% in 1968 [24]. Then later the magnitude of the peak HBsAg prevalence (10.8%) was available in 1986 and 1989 [25, 26] and then decreased to 6.2% in 2003 [27] and 5.3% in 2007 [28] from the community based studies (Fig. 2a). However, studies conducted in blood donors (Fig. 2b) reported a slightly higher median prevalence of 8.7% (IQR = 4.6–16.9) than the 6.2% median (IQR = 5.6–9.9) prevalence rate in the community based studies (Fig. 2a). Moreover, the reports from the blood donors of the same localities like in Bahir Dar (25 vs. 4.11%) [8, 13], Jimma (24.2 vs. 2.1%) [14, 15] and Gondar (14.4 vs. 4.7%) [16, 17] showed marked epidemiological discrepancies over time (Fig. 2b). The HBsAg was also reported among various segments of the society such as healthcare professionals (7.3–9.0%) [29, 30], medical waste handlers (6.0–6.3%) [31, 32], outpatient and inpatient department attendants (4.7–7.4%) [33, 34], street dwellers (10.9%) [35], pregnant women (3.0–7.3%) [36–40], diabetic patients (3.7%) [41], HIV VCT centres clients (5.7%) [10, 42] and commercial sex workers (6.0%) [43] (Fig. 2c). The HBsAg prevalence among Ethiopians Jews who immigrated to Israel in different times also showed 6.2 to 19% prevalence rate (11.5% median; IQR 7.6–16.6) (Fig. 2d). Overall, the median HBsAg prevalence in the general population (Fig. 2a-d) showed 6.3% (IQR = 5.2–10.8) over the last five decades.

**Fig. 3:**
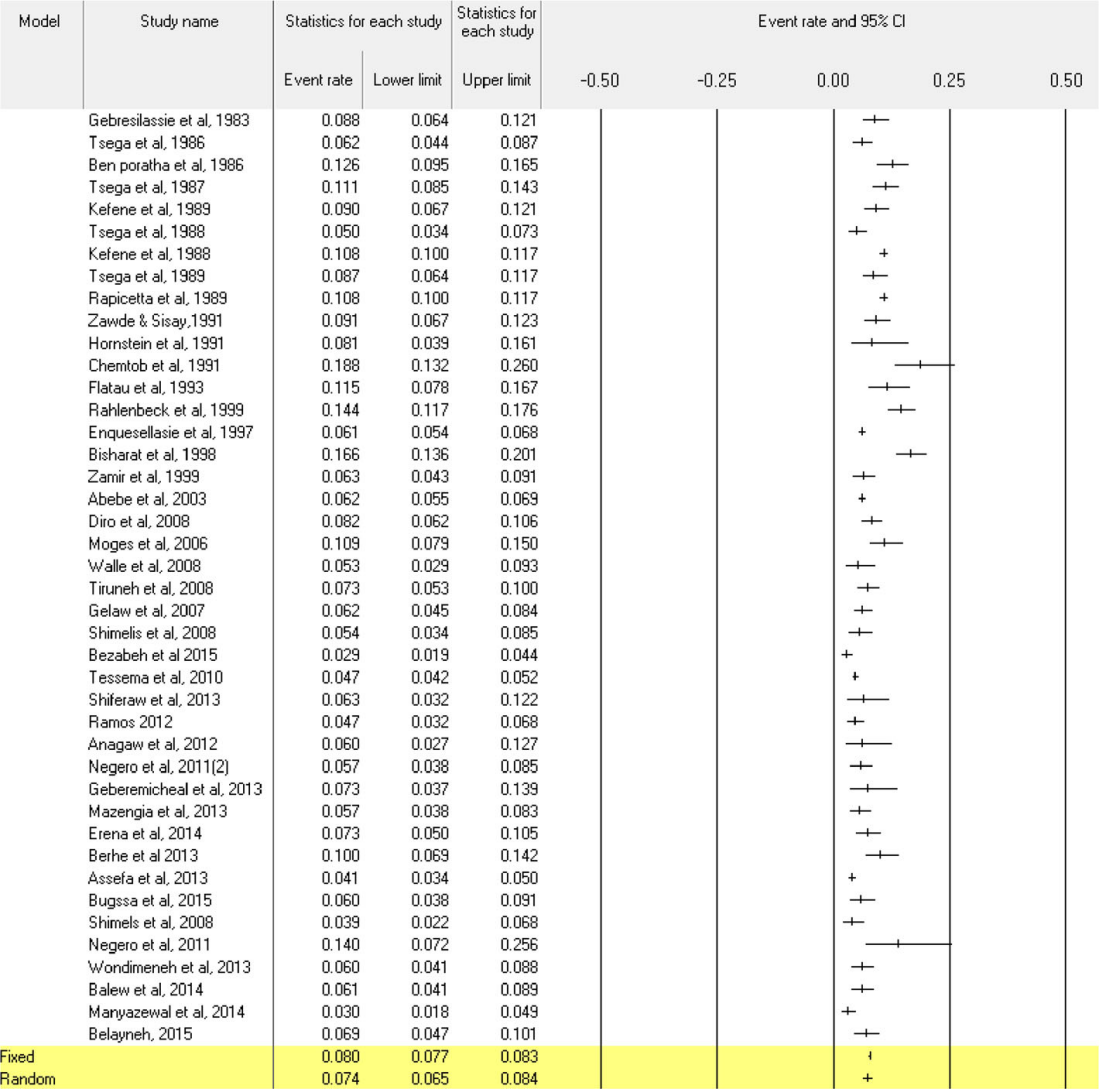
The meta-analysis and forest plot presentation of the HBsAg seroprevalence from 1983 to 2015. (Citations of studies used in the analysis from top to bottom: [9, 10, 13, 16, 17, 25–27, 29–35, 38, 39, 42, 43, 53, 54, 57, 59, 62–65, 67, 85, 87–90, 92, 93, 95–97])

Fourty two (67.7%) out of the 62 studies which reported HBsAg prevalence (Additional file 1) were used in the meta-analysis among the general population. The Cohran’s Q and I^2^ statistic for HBsAg was 509.7 and 91.9% (*p* < 0.001), respectively which showed the studies were heterogeneous. The overall pooled prevalence of HBV was 7.4% (95%CI: 6.5–8.4) (Fig. 3). The subgroup meta-analysis of study parameters such as the mean age group, study population types, year of study and geographical zones showed significant variations in the HBV prevalence (Table 1). In particular, the pooled prevalence among subgroup was 5.2% (95%CI: 3.7–7.4) in HIV infected individuals, 8.0% (95%CI: 5.9–10.7) in community based studies, 8.4% (95%CI: 5.4–12.7) in blood donors, 11.0% (95%CI: 7.5–15.9) in immigrants and 6.9% (95%CI: 5.6–8.5) in other groups. Among the above study parameters considered during meta-regression analysis, only study years were associated with a decreasing HBV prevalence over time (Additional file 3). The meta-regression plots for the mean age group and study years were presented in Additional file 4. Similarly, many studies in symptomatic liver disease patients reported HBsAg detection ranging from 9.9 to 35.8% [44–51] with a median prevalence of 21.2% (IQR = 13.4–33.6) (Table 2). The meta-analysis estimate among these patients also showed 20.0% (95%CI: 15.0–26.1) (Additional file 5).

**Table 1:** Subgroup meta-analysis of HBV and HCV prevalence estimation in Ethiopia

	Study parameters	Subgroup	Studies included	Prevalence %(95% CI)	I^2^%	*P*-value
HBV	Study population	Community based	7	8.0 (5.9–10.7)	96.1	<0.001
		Blood donors	6	8.4 (5.4–12.7)	95.4	<0.001
		HIV co-infected	7	5.2 (3.7–7.4)	75.9	<0.001
		Immigrants to Israel	5	11.0 (7.5–15.9)	79.0	0.001
		Other groups^a^	17	6.9 (5.6–8.5)	87.3	<0.001
	Mean age group^b^	14–29	8	7.1 (5.6–8.9)	85.3	<0.001
		30–45	9	6.7 (5.6–8.1)	39.6	<0.001
	Study year	1980–1989	9	9.4 (8.2–10.7)	74.5	<0.001
		1990–1999	8	10.7(7.3–15.4)	94.3	<0.001
		2000–2009	9	6.3 (5.1–7.9)	76.6	<0.001
		2010–2015	16	5.7 (4.9–6.6)	63.4	<0.001
	Geographical zone	Central Ethiopia	13	6.4 (5.3–7.6)	81.3	<0.001
		Northwest Ethiopia	17	8.2 (6.2–10.8)	93.7	<0.001
		Other regions^c^	12	7.7 (6.5–9.1)	82.6	<0.001
HCV	Study population	General population^d^	16	2.4 (1.6–3.6)	90.4	<0.001
		HIV co-infected	8	5.5 (3.8–7.8)	81.8	<0.001
	Mean age group	19–29	6	2.3 (0.9–5.8)	96.6	<0.001
		30–39	9	3.7 (2.4–5.8)	84.4	<0.001
	Study year	1990–1999	3	2.0 (1.5–2.7)	0.00	<0.001
		2000–2009	6	2.9 (1.4–5.9)	94.2	<0.001
		2010–2015	15	3.7 (2.5–5.4)	88.2	<0.001
	Geographical zone	Central Ethiopia	6	3.5(2.0–6.0)	89.0	<0.001
		Northwest Ethiopia	10	2.8(1.6–4.7)	87.8	<0.001
		Other regions^e^	8	3.4(1.7–6.5)	94.8	<0.001

^a^Other groups include: healthcare workers and medical waste handlers, out and inpatient department patients, street dwellers, pregnant women, diabetic patients, HIV voluntary counseling and testing (VCT) centers clients and commercial sex workers. ^b^It considered for those studies which only reported mean age

^c^Includes: Nationwide studies (*n* = 3), North (*n* = 1), North east (*n* = 2), South east (*n* = 5) and South west (*n* = 1). ^d^It includes: Community based studies, blood donors, HIV co-infected and immigrants. ^e^one, three, one, and three studies were from nationwide, north, northeast and southwest Ethiopia, respectively

**Table 2:** HBsAg prevalence of acute and chronic liver disease patients in different regions of Ethiopia from 1984 to 2013

Sampling year	Study area	Study design	Study Population	HBsAg, n (%)	Patients’ characterization and evaluation system^a^	References
1984	A.Ababa	Prospective longitudinal	304	30(9.9)	Patients with acute or chronic hepatitis, some with malignant liver disease, mainly hepatocellular carcinoma (HCC)	[45]
1989	A.Ababa	Consecutive hospital patients	334	91(27.2)	Based on clinical and histological diagnostic criteria; patients with chronic hepatitis (*n* = 114), cirrhosis (*n* = 208) and hepatocellular carcinoma (*n* = 112)	[46]
1991	North Ethiopia	Epidemic outbreak	423	54(12.8)	Icteric patients hospitalized during HEV	[47]
1992	A.Ababa	Consecutive hospital patients	110	22(20.0)	Consecutive acute hepatitis patients and 19 pregnant women who had HEV infection	[48]
1995	A.Ababa	Consecutive hospital patients	238	65(27.3)	Patients with chronic hepatitis (*n* = 14), cirrhotic (*n* = 156) and HCC (*n* = 68)	[49]
2011	A.Ababa	Prospective consecutive hospital patients	120	43(35.8)	Patients with chronic liver disease based on clinical history, ultrasound, and impaired liver function tests.	[44]
2011	Gondar	Retrospective	2684	386(14.4)	Five year retrospective study on clinically suspected hepatitis patients from different wards of Gondar hospital	[50]
2013	Bale Robe	Retrospective	358	80(22.3)	All patients with chronic hepatitis	[51]

^a^The clinical diagnostic criteria for grouping patients as chronic liver disease patients were based on the presence of patient history of either clinical signs like ascites, hepatomegaly, and splenomegaly with other clinical features including jaundice, palmar erythema, clubbing, edema, axillary and pubic hair loss, Spider nevi, flapping tremors, drowsiness, confusion and coma. Ultrasound showing coarse hepatic texture, changes in liver size, increased portal vein diameter, and splenomegaly. Impaired liver function tests, including raised level of alanine aminotransferase (ALT)

In some studies, along with HBsAg other HBV markers; anti-hepatitis B surface antigen (anti-HBs), anti-hepatitis B core antigen (anti-HBcAg) and hepatitis e Ag (HBeAg) and anti-hepatitis e antigen (anti-HBeAg) were also determined (Table 3). The peak prevalence rate (82%) for any markers was reported among chronic liver disease patients, whereas the lowest rate (42.2%) was in the general population [52]. The anti-HBs antibody was recorded at 11.6% in HIV infected individuals [53] and the highest rate (73.6%) was among healthcare workers [25]. Similarly, the HBeAg prevalence rate ranges from 0.6 to 44.0% (Table 3).

**Table 3:** Magnitude of HBV markers among different study populations in different regions of Ethiopia from 1983 to 2011

					Other HBV markers				References
Sampling year	Study area	Study groups	Sampled population	HBsAg n (%)	anti-HBs, n (%)	anti-HBcAg, n (%)	anti-HBeAg, n (%)	HBeAg, n (%)	
1983	Nationwide	Gen. population, children & ANC attendants	396	36 (9.1)	221 (67.2)^a^	-	19(55.9)^d^	15(44.0)^g^	[85]
1984	Nationwide	General population	500	31 (6.2)	152 (30.4)	16 (3.2)	10(32.3)^e^	8(25.8)^e^	[52]
1987	A. Ababa	Health workers	432	39 (9.02)	200 (46.3)	316 (73.1)	-	-	[29]
1989	Nationwide	General population	5265	569 (10.8)	61.4^b^	132 (2.5)	298(57.1)^f^	99(19.0)^f^	[26]
1994	A.Ababa	General population	4736	292 (6.2)	1796(40.7)^c^	1734 (36.6)	-	71/279 (25.4)	[27]
1995	A.Ababa	Chronic liver disease patients	238	65 (27.0)	131 (55.0)	-	-	-	[49]
1998	Immigrants to Israel	Familial members	506	84 (16.6%)	-	-	-	17/84(20.2)	[95]
2007	A. Ababa	HIV patients & VCT clients	620	29 (4.7)	-	277 (44.7)	-	3/29(10.3)	[42]
2010	Gamo HP	Inpatients department clients	556	26 (4.7)	-	7 (1.4)	-	-	[33]
2011	A.Ababa	HIV patients	500	15(3.0)	58(11.6)	-	-	3(0.6)	[53]

^a^ANC-Antenatal Care. ^b^Only the percentage was presented from the origional study. HBeAg and anti-HBeAg percentages were calculated on the basis of sample size of HBsAg positive individuals; ^e^ = 31, ^d^ = 34, ^f^ = 522 and ^g^ = 19. ^a^ = 329 and ^c^ = 4414 among HBsAg negatives

### Epidemiology of HCV

**Fig. 4:**
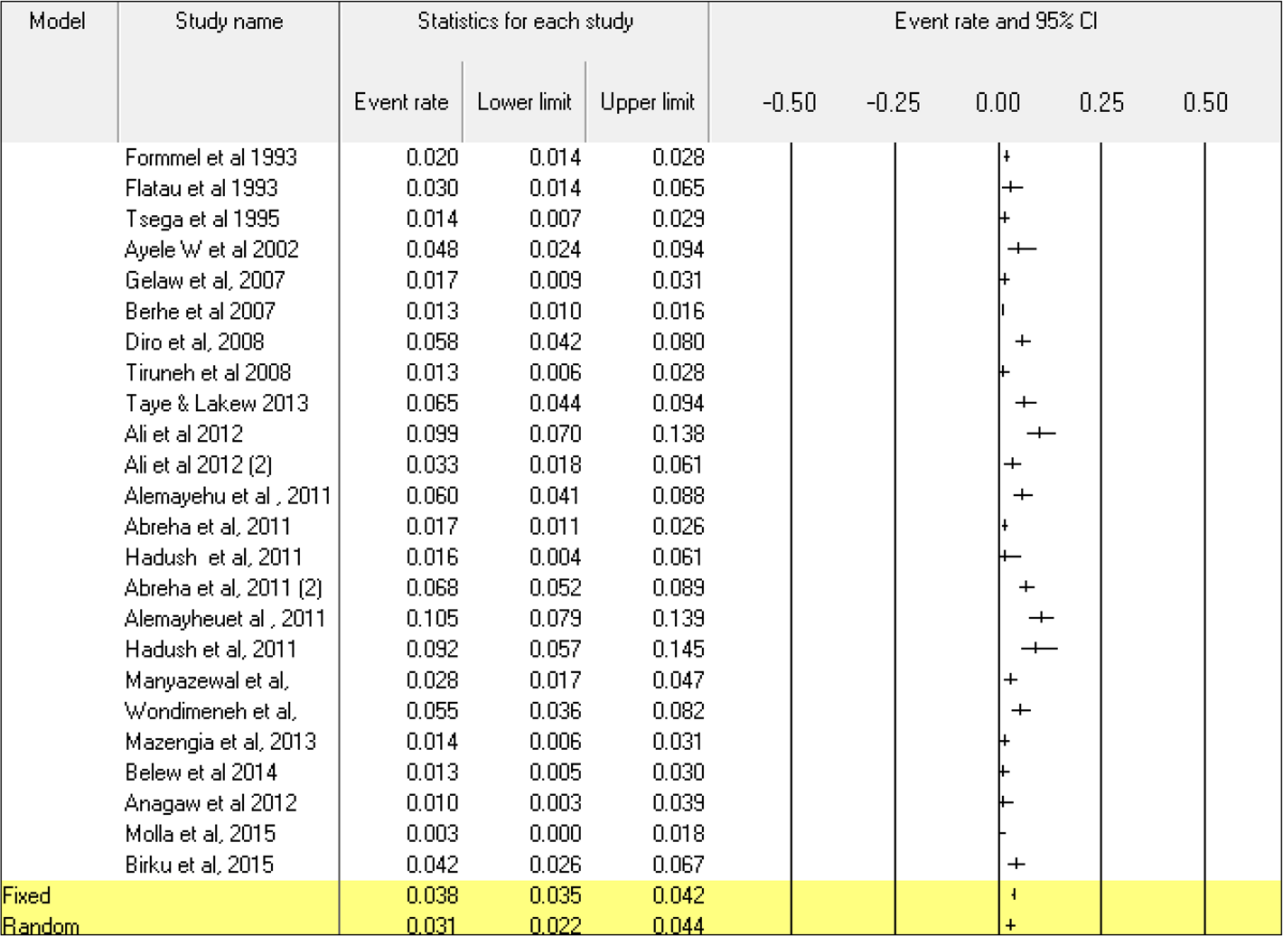
The meta-analysis and forest plot presentation of anti-HCV seroprevalence from 1993 to 2015. (Citations of studies used in the analysis from top to bottom: [9–12, 28, 32, 39, 49, 53–58, 64, 66–68, 98, 99])

Except a 3% prevalence rate of the time for Ethiopian immigrants to Israel in 1991 [54], the seroepidemiological survey of HCV in a community based studies showed 0.8 to 2% prevalence rate before the year 2000 [55, 56]. Over the years 2000s, relatively lower and comparable prevalence rates were documented from blood donors in north Ethiopia (1.7%) in 2007 [57], Gondar (0.7%) in 2010 [17], Jimma (0.2%) [16] and Bahir Dar (0.6%) in 2011 [13] (Table 4). In contrast, unusually high HCV prevalence was reported in blood donors from Gondar (5.8%) in 2008 [9] and from Bahir Dar (13.3%) in 2007 [8]. A similar variation was also reported, among other groups, such as VCT clients [10–12], diabetic patients [58], antenatal care (ANC) [39] and medical waste handlers [32] (Table 4). Likewise, the magnitude of anti-HCV antibody from chronic liver disease patients showed a variable prevalence rate over time with high (37.8%) in 1995 [49] and low (2.7%) in 2014 [51] (Table 4). For the HCV meta-analysis, 24 (75.0%) studies were eligible and selected from the total of 32 studies (Additional file 2). The Cohran’s Q (283.1) and I^2^ statistics (91.9%, *p* < 0.001) showed heterogeneity among studies. The pooled prevalence rate of anti-HCV antibody was 3.1% (95%CI: 2.2–4.4) (Fig. 4). The subgroup meta-analysis showed significant variations in the HCV prevalence among the study parameters (Table 1).

**Table 4:** Magnitude of HCV antibody prevalence among different study population in different regions of Ethiopia from 1988 to 2014

Study group	Sampling year	Study area	Study population	Anti-HCV Ab frequency (%)	References
Community based	1993	A.Ababa & its outskirts	1580	32 (2.0)	[56]
	1994	A.Ababa	2663	29 (0.8)	[55]
	2002	Kemissie & Omo	5248	68 (1.3)	[28]
Blood donors	1995	A.Ababa	500	7 (1.4)	[49]
	2003	North Ethiopia	600	10 (1.7)	[57]
	2004	Gondar	600	35 (5.8)	[9]
	2006	Bahirdar	324	43 (13.3)	[8]
	2007	Gondar	6361	35 (0.7)	[17]
	2008	Bahir Dar	2384	15 (0.6)	[13]
	2010	Jimma	6063	12 (0.2)	[15]
Symptomatic liver disease patients	1988	North Ethiopia	139	20 (14.0)	[47]
	1992	A.Ababa	110	21 (19.0)	[48]
	1995	A.Ababa	238	90 (37.8)	[49]
	2011	A. Ababa	120	27 (22.5)	[44]
	2011	Gondar	2684	322 (12.0)	[50]
	2013	Bale Robe	220	6 (2.7)	[51]
HIV infected individuals	1994	A. Ababa	165	8(4.8)	[55]
	2006	A. Ababa	734	50 (6.8)	[66]
	2008	Hawassa	400	42(10.5)	[11]
	2011	A. Ababa	500	18 (3.6)	[53]
	2011	Mekele	174	16(9.2)	[12]
	2012	D/ Tabour	395	5(1.3)	[67]
	2013	A.Ababa	387	25(6.5)	[68]
	2013	Gondar	400	22(5.5)	[64]
	2013	Bahirdar	269	59(21.9)^a^	[100]
	2014	Bahirdar	253	14(5.5)	[69]
Others^b^	2006	Gondar	480	6 (1.3)	[39]
	2006	A. Ababa	1220	21 (1.7)	[66]
	2008	Hawassa	400	24(6.0)	[11]
	2010	Jimma	304	30 (9.9)	[58]
	2010	Jimma	300	10 (3.3)	[58]
	2010	Gamo HP	556	1 (0.2)	[33]
	2011	Gondar	200	2 (1.0)	[32]
	2011	Mekele	126	2 (1.6)	[12]
	2012	D/ Markos	423	6 (1.4)	[10]

^a^Reported as 18.9% in the original work, but the 3.0% anti-HCV antibody reported as co-infected with HBsAg was summed up and became 21.9%

^b^Includes antenatal care attendants, HIV voluntary counselling and testing (VCT) clients (HIV negative), VCT clients, diabetic patients, non-diabetic patients (controls for diabetic patients), out and inpatient department clients, healthcare workers and medical waste handlers, and VCT clients

### Epidemiology of HAV, HDV and HEV

An early report of HAV antibody prevalence rates was 84 and 50% [46, 59], respectively among the general population and children. In regard to HDV, available scientific reports in the 1980s showed from 5.8 to 9.6% of anti-HDV antibody prevalence rates among the general population [59, 60]. HEV was also reported 59% in acute pregnant women [61] and 93% in acute patients and 3% in healthy controls [47].

### Epidemiology of viral hepatitis among HIV infected individuals

Cases-control studies of HBsAg among HIV infected individuals showed a record of 14% (vs. 4.3% in the control) in Shashemene (Southern Ethiopia) [62] and 5.4% (vs. 3.9% in the controls) from the central Ethiopia in Addis Ababa [42]. Studies which included HIV infected individuals alone reported 2.3% HBsAg prevalence rate in Woldiya [63] and 5.6% in Gondar [64] and 6.9% in Hawassa [65] from the north, northwest and south part of Ethiopia, respectively. Overall, for the last 10 years, the median HBsAg seroprevalence among HIV infected individuals was 6.0% (IQR = 3.0–6.0) with an increased trend prevalence (*X* ^2^ = 15.1, *p* < 0.001) when compared to HIV negative controls. In the subgroup meta-analysis, HBV prevalence in HIV co-infected individuals was 5.2% (95% CI 3.7–7.4, *p* < 0.001) (Table 1).

The prevalence rate of HCV antibody in HIV infected individuals was 4.5%, which was higher than HIV negative controls (0.8%) in Addis Ababa [55]. A study from southern Ethiopia in Hawassa [11] showed a 10.5% prevalence of HCV in HIV infected individuals compared to 6.0% in the HIV negative control group. In this report and another similar study in Addis Ababa [65], HCV viraemia was reported 9.1 and 25.4%, respectively from anti-HCV antibody positive individuals. Overall, the HCV prevalence showed an increasing trend prevalence (*X* ^2^ = 18.7, *p* < 0.001) in HIV infected individuals than non-infected counterparts with a median prevalence of 6.5% (IQR = 4.2–9.9) [11, 12, 53, 55, 64, 66–69]. In the subgroup meta-analysis, the prevalence of anti-HCV antibody in HIV co-infected individuals was 5.5% (95% CI 3.8-7.8, *p* < 0.001), respectively (Table 1 and Additional file 4). Unlike mean age, study year and geographical zones, the meta-regression analysis showed that HIV co-infection was associated with higher HCV prevalence rate (Additional files 3 and 4).

## Discussion

This is the first detailed systematic review and meta-analysis study of viral hepatitis in Ethiopia over a large period of time. The findings clearly show the burden of viral hepatitis in the country in general and endemicity of HBV in particular. The findings were also consistent with the estimate of 5–20% of most African countries [70] and the WHO HBV endemic definition (5–7% HBsAg prevalence) in the general population of a defined geographical area [1]. Similarly, HBV markers other than HBsAg ever reported in the country have been in agreement with data from most African countries and support the endemicity of HBV estimated from the HBsAg prevalence.

HBV seroprevalence studies in blood donors were merely geographical representative, which covered north, northwest, southwest and central parts of Ethiopia. However, the HBsAg prevalence in this group was a bit higher (8.4%) than the community based studies (8.0%), but in agreement with reports on blood donors of other African countries [70–72]. Nevertheless, in Ethiopia, studies in this group showed a marked variation even in the same locality with exceptionally highest [8, 14] or lowest [13, 15] prevalence rates over time. Apart from geographical location and study time differences, the discrepancies in this group in particular and other groups in general could be due to methodological drawbacks such as small sample size seen from southwest Ethiopia [14] and ill-defined study design from northwest Ethiopia [50]. For instance, the latter study failed to exclude patients who were screened HBsAg for differential diagnosis of other illness, but rather reported 14.4% HBsAg prevalence by considering the subject as ‘clinically suspected viral hepatitis patients’. The other source of the discrepancy might be due to the potential variability in sensitivity and specificity of the commercially available test kits used in each study, although the studies used in this review were based on an immunoassay based screening kits with more or less similar principle of antibody detection.

From early 1980s to the present time, more than 120,000 [73] Ethiopian Jews immigrated to Israel. Because of the low standards of socio-economic and healthcare status of these migrants, they attracted the focus of public health intervention and were screened for infectious disease agents including hepatitis viruses [74, 75]. The 11.0% HBsAg prevalence rate observed were higher than the blood donors, the general population and other groups. This could be due to the fact that the immigrates were originally from rural Ethiopians communities where risky traditional unsafe practices (tattooing, ear piercing, tonsillectomy, circumcision, ritual scars, traditional surgery, unsterilised shaving at the barber shop, dental extraction at home, home delivery by traditional birth attendants, unsafe abortion practices) are commonly practiced. The finding in this group, however, could represent and reflect HBV prevalence in the rural settings of Ethiopia since the majority of the studies concentrated in urban settings. Moreover, the prevalence observed in these immigrants also could indirectly reflect the potential distribution dynamics of hepatitis viruses to the less endemic parts of the world.

In Ethiopia, reports on HBsAg prevalence were also common from groups such as pregnant women, diabetic patients, street dwellers, HIV VCT clients and commercial sex workers. The HBsAg prevalence was hyperendemic among healthcare professionals [29] and medical waste handlers [30] in particular might be associated with occupational risk exposures since they deal with all sorts of the infected samples with no HBV vaccination, which is the simplest protection and standard practice elsewhere. A recent study showed that although knowledge about availability of HBV vaccine was 62% among healthcare workers, fully vaccinated professionals were only 5.4% [76]. Another study conducted on Ethiopian surgeons showed that most of them were not vaccinated because of negligence and lack of knowledge about the recent introduction of the vaccine, despite their strong belief HBV vaccine is useful [77]. This could explain the reason that the relatively higher prevalence rate of HBsAg and anti-HBS antibody level among healthcare workers in Ethiopia since the exposure and subsequent natural immune development could be expected relatively higher in this group. As the result, to minimize at least an occupational exposure of HBV infection for such risky groups, an urgent need for the HBV vaccination scheme, awareness creation and attitudinal health education on the universal precaution and safety of blood born infection transmissions are crucial in Ethiopia.

The disease burden caused by HBV and other forms of hepatitis is undocumented in Ethiopia, although acute viral hepatitis, chronic hepatitis, cirrhosis of the liver and HCC accounted for significant hospital admissions and mortality rate [78]. In late 1990s, the most common tumour in medical units in Ethiopia was associated with a 50% carrier rate of HBsAg [7, 78]. In line to this, the overall HBsAg prevalence in the symptomatic liver disease patients estimated 20.0 and 21.2% from the meta-analysis and systematic review analysis, respectively.

Regarding HCV, the prevalence rate in different time zones was estimated in Ethiopia before as 1.9% [79], 1.3% [80] and 2.7% [81] as part of the HCV epidemiological estimation in sub Saharan African countries. However, the above estimates were limited in terms of numbers of studies included, study time, study groups and geographical representation of the studies available throughout the country. For instance, the former two estimates were made from four studies [9, 17, 28, 56] in which two of them were from blood donors of the same study area. However, in the current study, a comprehensive estimate analysis was made from three groups independently analysed as the general population (includes community based, blood donors, healthcare workers, etc.), symptomatic liver disease patients and HIV infected individuals representing a vast geographical area of the country and time. The overall 3.1% pooled prevalence estimate and the magnitude of anti-HCV antibody from 2.7 to 37.8% in chronic liver disease patients [44, 49], therefore, might show the true epidemiological picture and the real burden of HCV in Ethiopia. This is also supported by the level of HCV viraemia reported in 25.4% of HCV antibody positive individuals [66]. The pooled prevalence in this study was higher than the recent reports from neighbouring countries of 0.3% in Djibouti, 0.9% in Somalia, and 1.0% in Sudan [82] but in line with a 3.0% prevalence estimate of most other African countries [79, 83]. The risk factors discussed for transmission of HBV in Ethiopia also might play their own roles in the transmission of HCV, although the degree of transmission potential varies for both viruses.

Similarly, unlike HBV and HCV, prevalence studies of HAV, HDV and HEV among the general population and liver disease patients were very few in Ethiopia. The data available so far [47, 60, 61, 84] showed a considerate prevalence particularly for HAV and HEV infections. These viruses are transmitted by the faecal-oral route and many of the environmental and socio-economic factors foster the transmission routes. In Ethiopia, the exposure to HAV at least once in every individual was common and the prevalence rates range from eight to 99% in the general population [48, 85]. However, unlike the blood born viral hepatitis, HAV and HEV do not commonly exist in a chronic carrier state [84] and they are very different in exposures/transmissions. In Africa, HEV infection is widespread and is expected to be a threat to numerous lives, especially for those pregnant women and their foetuses [4]. A serological data from Egypt showed that the seroprevalence of anti-HEV can reach close to 100% in the general population [4]; similar to anti-HAV prevalence in Ethiopia and elsewhere in Africa [84, 86]. It was also reported that in many countries like Sudan, Chad, Uganda, Kenya, Somalia and Ethiopia a number of large HEV outbreaks occurred at different times [4]. For instance, in Uganda alone, the mortality rate among children younger than 2 years of age was 8% [4]. Similarly, in Ethiopia, the HEV antibody seroprevalence observed in jaundice patients was 93% [48] but the least from acute sporadic patients (33%) [48], pregnant women (59%) and healthy adults (3%) [48]. According to this report, in the early 1990s, HEV was a common cause of acute sporadic viral hepatitis in Ethiopian patients. In particular, the occurrence during pregnancy was associated with high maternal and foetal morbidity and mortality [48]. Although there is no current study available to compare, the late 1980s HDV reports ranged from 5.8 to 9.6% could classify Ethiopia as one of an endemic country. The worldwide estimation also indicated that 5% of HBsAg carriers were infected with HDV [5]. In Ethiopia, an old report showed that the distribution of HBV markers was similar to anti-HDV positive and anti-HDV negative individuals due to the relatively young age of the population and/or hyperendemic condition of the area [60].

Although an increasing pattern of viral hepatitis in general is predictable from HIV infected individuals in Ethiopia, unlike HCV (5.5%), the co-prevalence estimate of HBV (5.2%) with HIV were lower than the estimate of other groups. Similarly, the meta-regression analysis showed a decreasing pattern of overall HBsAg prevalence rate over study year. The intensive prevention and control measures done after the era of HIV pandemic might be responsible for HBV exposure reduction. Therefore, this could might encourage a need for integrating HBV management to the HIV prevention and control programs in Ethiopia. However, unlike HBV, the HCV seroprevalence analysis in the HIV infected individuals was higher up to two folds than HIV negative controls. Regarding the HAV, HDV and/or HEV co-infections with HIV in Ethiopia, there are no data available to compare with mono-infection of each hepatitis virus.

## Conclusion

Given the potential limitations arising from small sample size study effects, inconsistency of the screening kits used in the studies as well as study design, time and area heterogeneity, the findings from the systematic review and meta-analysis were in agreement with each other and indicates all forms of viral hepatitis infection are common in Ethiopia. In particular, HBV and HCV and/or their respective co-infection with HIV are prevalent and endemic. These could be taken as one of the nation’s public health problems and potential healthcare threats especially in compromising HIV management. Recent studies on the magnitude of HAV, HDV and HEV are lacking, hence, the need for further studies to generate up-to-date data is recommended. Developing viral hepatitis diagnosis algorithms, treatment schemes and an enhanced HBV vaccination program for children and risk groups in Ethiopia are strongly recommended. The HBV/HCV co-prevalence with HIV supports the need for preventive measures and screening of viral hepatitis in Ethiopia and could be integrated into the country’s existing HIV prevention and control programs.

## Additional files

Additional file 1: The major characteristics of studies which reported HBV seroprevalence in Ethiopia. Description of data: Abbreviations; CE-Central Ethiopia, NE-North Ethiopia,NW- Northwest Ethiopia, SE-South Ethiopia, SW-Southwest Ethiopia. CS-Cross sectional, RS-Retrospective study, PC-Prospective Cross sectional, PL-Prospective longitudinal study. EIA: Enzyme immunoassay, ELISA: Enzyme linked immunoassay, RIA: Radioimmunoassay, CIA-Chromatographic immunoassay. ANC-antenatal care, MWHs/NMWHs-medical waste handlers / Non-medical waste handlers, CLD-Chronic liver disease. ^+^DNA Hybridization. ** (A = 9–12), (B =5–8), (C = 1–4). ^¥^For Meta-analysis. Blank cells in the table indicated that the information was unavailable in the original articles. (DOCX 102 kb)
Additional file 2: The major characteristics of studies which reported HCV seroprevalence in Ethiopia. Description of data: Abbreviations; CE-Central Ethiopia, NE-North Ethiopia,NW- Northwest Ethiopia, SE-South Ethiopia, SW-Southwest Ethiopia. CS-Cross sectional, RS-Retrospective study, PC-Prospective Cross sectional, PL-Prospective longitudinal study. EIA: Enzyme immunoassay, ELISA: Enzyme linked immunoassay, RIA: Radioimmunoassay, CIA-Chromatographic immunoassay. ANC-antenatal care, MWHs/NMWHs-medical waste handlers / Non-medical waste handlers, CLD-Chronic liver disease. ^+^DNA Hybridization. ** (A = 9–12), (B =5–8), (C = 1–4). ^¥^For the meta-analysis. Blank cells in the table indicated that the information was unavailable in the original articles. Different quality scores might be observed in a single study which reported both HBV and HCV simultaneously. (DOCX 70 kb)
Additional file 3: Results of meta-regression analysis of HBV and HCV prevalence. (DOCX 27 kb)
Additional file 4: Regression plot for mean age and study year to HBV (Fig. A and B) and HCV (Fig. C and D) prevalence. (TIF 625 kb)
Additional file 5: The meta-analysis and forest plot presentation of the HBsAg prevalence in the liver disease patients from 1984 to 2014. (TIF 145 kb)

## Abbreviations

ANC: Antenatal care
Anti-HBcAg: Anti-hepatitis B core antigen
Anti-HBeAg: Anti-hepatitis e antigen
Anti-HBs: Anti-hepatitis B surface antigen
Anti-HCV: Anti-hepatitis C virus antibody
EIA: Enzyme immunoassay
ELISA: Enzyme linked immunoassay
HAV: Hepatitis virus
HBeAg: Hepatitis e Ag
HBsAg: Hepatitis B surface antigen
HBV: Hepatitis B virus
HCC: Hepatocellular carcinoma
HCV: Hepatitis C virus
HDV: Hepatitis D virus
HEV: Hepatitis E virus
HIV: Human immunodeficiency virus
I^2^: Inconsistency statistics
IQR: Inter quartile range
PRISMA: Preferred Reporting Items for Systematic Reviews and Meta-analysis
RIA: Radioimmunoassay
VCT: HIV voluntary counselling and testing
WHO: World Health Organization
