# Erratum to: Hepatitis viruses in Ethiopia: a systematic review and meta-analysis

**DOI:** 10.1186/s12879-017-2181-7

**Published:** 2017-02-01

**Authors:** Yeshambel Belyhun, Melanie Maier, Andargachew Mulu, Ermias Diro, Uwe Gerd Liebert

**Affiliations:** 1 Institute of Virology, Faculty of Medicine, Leipzig University, Leipzig, Germany; 2 School of Biomedical and Laboratory Sciences, College of Medicine and Health Sciences, University of Gondar, Gondar, Ethiopia; 3 Department of Internal Medicine, College of Medicine and Health Sciences, University of Gondar, Gondar, Ethiopia

## Erratum

After the publication of this work [1] it was noticed that the citation lists on figures 3 and 4 were incorrect. The original version of this article was corrected.

The citation list on figure 3 was put as:

[9, 10, 13, 16, 17, 25–27, 29–35, 38, 39, 42, 43, 53, 54, 57, 59, 62–65, 67, 85, 87–90, 92, 93, 95–97]

It should have been:

[85, 59, 91, 89, 29, 87, 25, 87, 26, 90, 92, 93, 54, 16, 88, 95, 96, 27, 9, 35, 38, 39, 57, 42, 97, 17, 31, 33, 32, 62, 30, 10, 34, 63, 13, 43, 42, 62, 64, 67, 53, 65]

The citation list on figure 4 was put as:

[9–12, 28, 32, 39, 49, 53–58, 64, 66–68, 98, 99]

It should have been:

[56, 54, 49, 55, 57, 28, 9, 39, 68, 58, 58, 11, 66, 12, 66, 11, 12, 53, 64, 10, 67, 32, 98, 99]

The publisher apologises for these errors.
