# Supplementary material for: Hepatitis viruses in Ethiopia: a systematic review and meta-analysis
Source: BMC Infect Dis. 2016 Dec 19;16:761. doi: 10.1186/s12879-016-2090-1 (PMC5168848; doi:10.1186/s12879-016-2090-1)
Supplement: Additional file 1: — The major characteristics of studies which reported HBV seroprevalence in Ethiopia. Description of data: Abbreviations; CE-Central Ethiopia, NE-North Ethiopia,NW- Northwest Ethiopia, SE-South Ethiopia, SW-Southwest Ethiopia. CS-Cross sectional, RS-Retrospective study, PC-Prospective Cross sectional, PL-Prospective longitudinal study. EIA: Enzyme immunoassay, ELISA: Enzyme linked immunoassay, RIA: Radioimmunoassay, CIA-Chromatographic immunoassay. ANC-antenatal care, MWHs/NMWHs-medical waste handlers / Non-medical waste handlers, CLD-Chronic liver disease. +DNA Hybridization. ** (A = 9–12), (B =5–8), (C = 1–4). ¥For Meta-analysis. Blank cells in the table indicated that the information was unavailable in the original articles. (DOCX 102 kb) [file 12879_2016_2090_MOESM1_ESM.docx]

Additional file 1

Additional file 1... (Continued)

|  |  |  |  |  |
| --- | --- | --- | --- | --- |
