# Supplementary material for: Hepatitis viruses in Ethiopia: a systematic review and meta-analysis
Source: BMC Infect Dis. 2016 Dec 19;16:761. doi: 10.1186/s12879-016-2090-1 (PMC5168848; doi:10.1186/s12879-016-2090-1)
Supplement: Additional file 3: — Results of meta-regression analysis of HBV and HCV prevalence. (DOCX 27 kb) [file 12879_2016_2090_MOESM3_ESM.docx]

Additional file 3

|  | **Study parameters** | **Slope coefficient** | **R^2^** | **P-value** |
| --- | --- | --- | --- | --- |
| **HBV** | **Mean age group^**^** | -0.009 | 0.00 | 0.57 |
|  | **Study year** | -0.025 | 0.57 | **<0.001** |
|  | **Study population** |  | 0.00 | 0.07 |
|  | Community based (Ref.) |  |  |  |
|  | Blood donors | 0.053 |  |  |
|  | HIV co-infected | -0.456 |  |  |
|  | Immigrants to Israel | 0.354 |  |  |
|  | Other groups* | -0.156 |  |  |
|  | **Geographical zone** |  | 0.11 | 0.19 |
|  | Central Ethiopia (Ref.) |  |  |  |
|  | Northwest Ethiopia | 0.298 |  |  |
|  | Other regions^£^ | 0.208 |  |  |
|  | **Study parameters** | **Slope coefficient** | **R^2^** | **P-value** |
| **HCV** | **Mean age** | 0.051 | 0.18 | 0.24 |
|  | **Study year** | 0.021 | 0.09 | 0.46 |
|  | **Study population** |  | 0.28 | **0.01** |
|  | Community based (Ref.) |  |  |  |
|  | HIV co-infected | 0.82 |  |  |
|  | **Geographical zone** |  | 0.00 | 0.82 |
|  | Central Ethiopia (Ref.) |  |  |  |
|  | Northwest Ethiopia | -0.024 |  |  |
|  | Other regions^£^ | -0.010 |  |  |
