# Supplementary figures and images for: Hepatitis viruses in Ethiopia: a systematic review and meta-analysis
Source: BMC Infect Dis. 2016 Dec 19;16:761. doi: 10.1186/s12879-016-2090-1 (PMC5168848; doi:10.1186/s12879-016-2090-1)

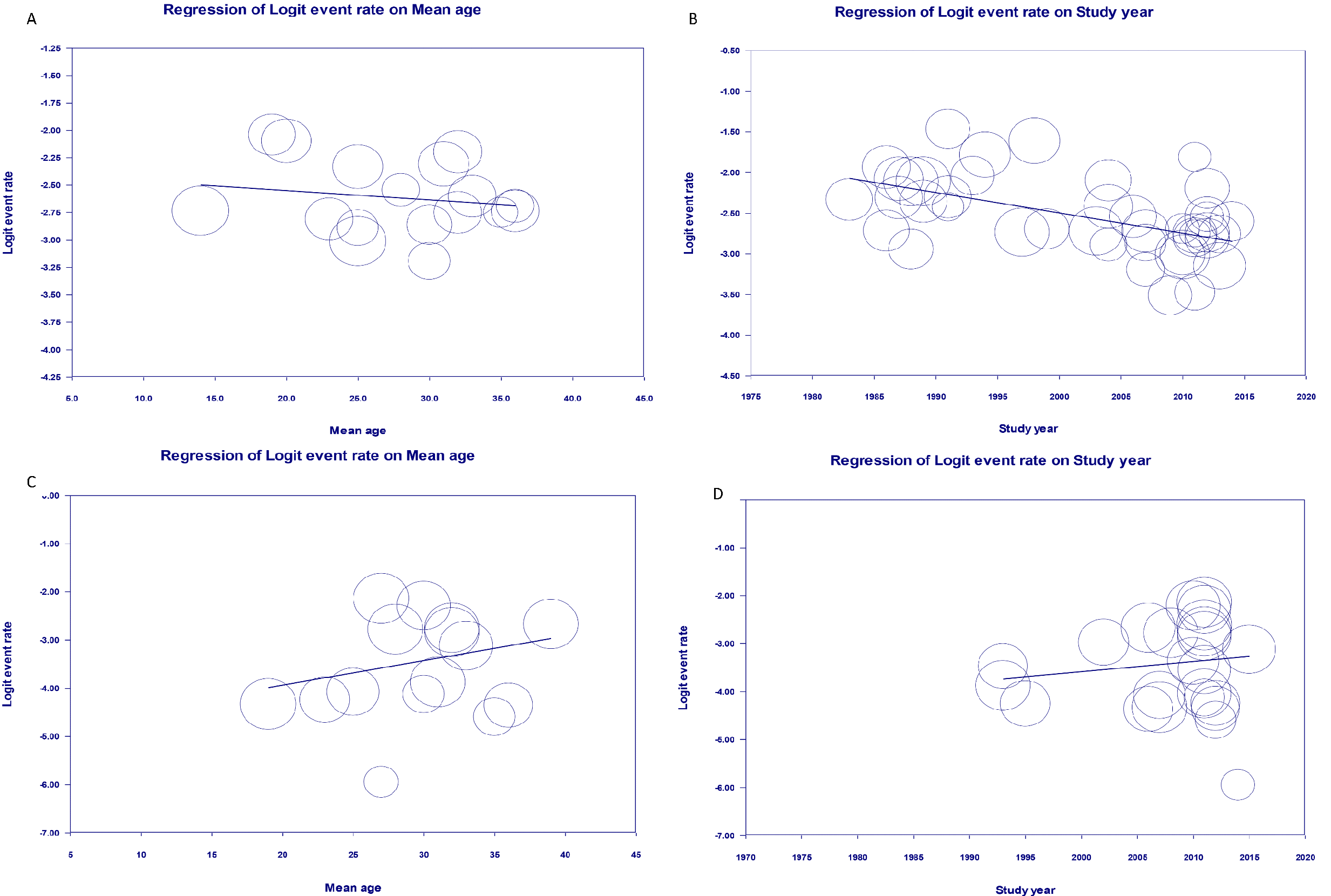

Supplement: Additional file 4: — Regression plot for mean age and study year to HBV (Fig. A and B) and HCV (Fig. C and D) prevalence. (TIF 625 kb) [file 12879_2016_2090_MOESM4_ESM.tif]

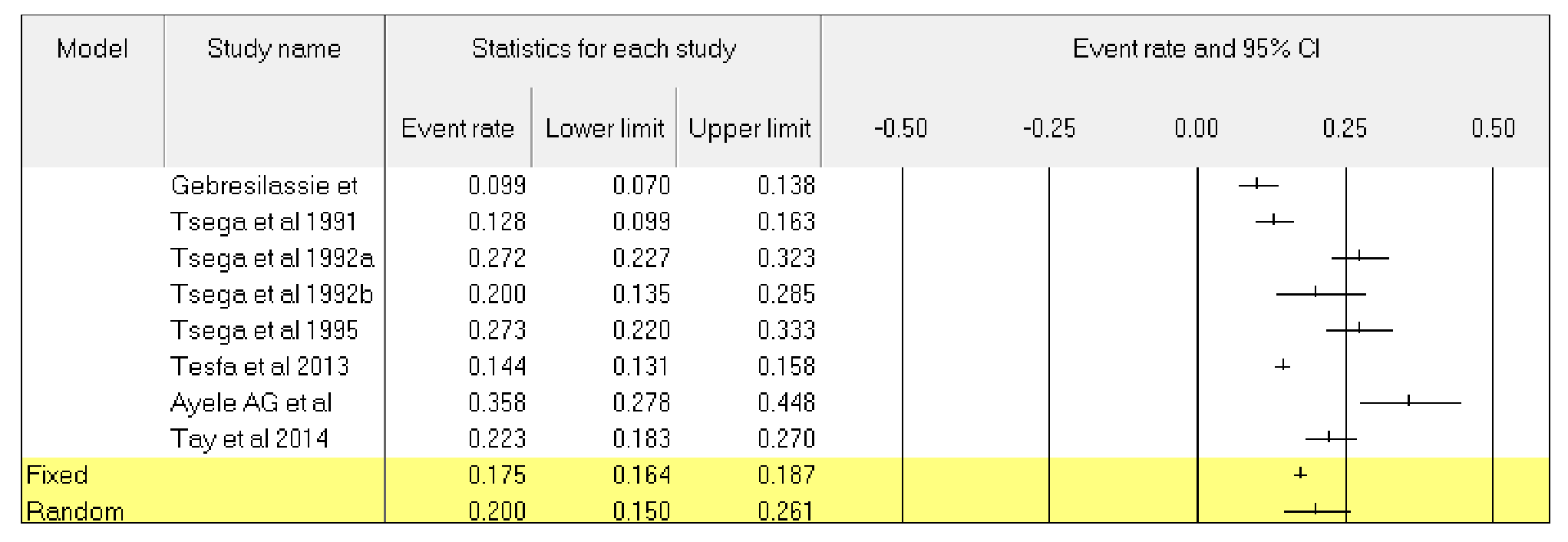

Supplement: Additional file 5: — The meta-analysis and forest plot presentation of the HBsAg prevalence in the liver disease patients from 1984 to 2014. (TIF 145 kb) [file 12879_2016_2090_MOESM5_ESM.tif]
